# Speed hysteresis and noise shaping of traveling fronts in neural fields: role of local circuitry and nonlocal connectivity

**DOI:** 10.1038/srep39611

**Published:** 2017-01-03

**Authors:** Cristiano Capone, Maurizio Mattia

**Affiliations:** 1 PhD Program in Physics and Department of Physics, Sapienza University, Rome, Italy; 2 Department of Technologies and Health, Istituto Superiore di Sanità, Rome, Italy

## Abstract

Neural field models are powerful tools to investigate the richness of spatiotemporal activity patterns like waves and bumps, emerging from the cerebral cortex. Understanding how spontaneous and evoked activity is related to the structure of underlying networks is of central interest to unfold how information is processed by these systems. Here we focus on the interplay between local properties like input-output gain function and recurrent synaptic self-excitation of cortical modules, and nonlocal intermodular synaptic couplings yielding to define a multiscale neural field. In this framework, we work out analytic expressions for the wave speed and the stochastic diffusion of propagating fronts uncovering the existence of an optimal balance between local and nonlocal connectivity which minimizes the fluctuations of the activation front propagation. Incorporating an activity-dependent adaptation of local excitability further highlights the independent role that local and nonlocal connectivity play in modulating the speed of propagation of the activation and silencing wavefronts, respectively. Inhomogeneities in space of local excitability give raise to a novel hysteresis phenomenon such that the speed of waves traveling in opposite directions display different velocities in the same location. Taken together these results provide insights on the multiscale organization of brain slow-waves measured during deep sleep and anesthesia.

Front propagation and interface motion occur in many scientific areas such as chemical kinetics, pattern formation, spread of epidemics and so on ref. 1. Front propagation has been studied in many theoretical contexts such as reaction and diffusion equations^1^,^2^,^3^ and neural field theory^4^,^5^. In experimental neuroscience, traveling waves are receiving increasing interest, as multielectrode recordings and optical imaging with calcium- and voltage-sensitive dyes allow a detailed characterization^6^,^7^,^8^. As a result, activation waves appear to be a collective phenomenon supported by the interplay between lateral synaptic interactions and the intrinsic input-output properties of local neuronal circuitry^9^,^10^,^11^,^12^,^13^. This multiscale organization of cortical waves calls for understanding the theoretical characterization of the phenomenon making elusive the interplay between network structure and spatiotemporal patterns of activity expressed^14^,^15^. Unraveling the relationship between structure and function is crucial to address questions of wide interest, like the mechanistic roots of neurological disorders which are known to affect ongoing rhythmic wave production in sleeping and anesthetized brains^16^,^17^.

Here we ask how does the multiscale structure of the cerebral cortex affect wave propagation. The proposed theoretical framework models the cortical tissue as an excitable medium which is continuum in space^4^,^5^, seen as a linear chain of neuronal assemblies each described by an equation introduced in the seminal work by Shun-ichi Amari^18^,^19^,^20^


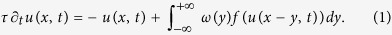


Under mean-field approximation, *u*(*x, t*) is the input current at time *t* to a neuronal population located in *x*. This input filters a linear combination of presynaptic activity *f*(*u*(*x, t*)) shaped by the connectivity kernel *ω*(*y*). *f*(*u*) is a nonlinear input-output gain function and low-pass filtering models a non-instantaneous synaptic transmission with decay *τ* = 1, here used as unit of time. Stable wavefronts can be worked out resorting to the Green’s function *η*(*t*) = *e*^−*t*/*τ*^*θ*(*t*) of the operator 
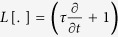
 and by defining *ξ* = *x* − *ct*, where *c* is the wave speed^4^,^5^. The input wavefront *q*(*ξ*) = *u*(*x* − *ct*) then results to be self-consistently defined as


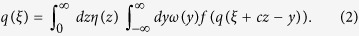


Relatively simple solutions can be found by considering a stepwise gain function *f*(*u*) = Θ(*u* − *h*) with input threshold *h*, and an exponential decaying connectivity *ω*(*y*) = *e*^−|*y*|/*R*^/2*R*, where *R* is the spatial extent of the kernel used here as length unit (*R* = 1)^4^,^5^.

Starting from this, we extend this minimal rate model by introducing a suitable piecewise linear gain function *f*(*u*). This allows to develop a perturbative approach to the analytic characterization of deterministic and stochastic propagation of wavefronts. In order to have a more accurate description of the experiments on cortical slow-wave activity, we finally incorporate three additional ingredients invariantly expressed in this framework by neuronal networks: i) an activity-dependent fatigue mechanism modeling neuronal spike frequency adaptation in active states, ii) a diverse local excitability in space for instance due to the different cytoarchitectonic organization of layers and cortical areas and iii) an additive white noise mimicking the endogenous activity fluctuations due to the finite size of cell assemblies.

## Results

### Impact of local excitability on wave speed

**Figure 1:**
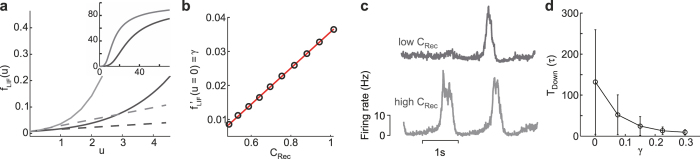
Local input-output gain function modulates Down state stability. (**a**) Sigmoidal current-to-rate gain function *f*_*LIF*_(*u*) of a homogenous recurrent network of excitatory leaky integrate-and-fire (LIF) neurons with relatively large and small (light and dark gray, respectively) connectivity levels *C*_*Rec*_. Dashed lines, gain function slopes around the quiescent state (*u* = 0). Inset, zoomed out *f*_*LIF*_(*u*). (**b**) Correlation between local connectivity *C*_*Rec*_ and the derivative of the gain function in *u* = 0. (**c**) Firing rate traces from LIF neuron networks with relatively large and small connectivity as in (a) (light and dark gray, respectively). Model networks are the same as in ref. 26. (**d**) Average residence times *T*_Down_ in the Down state for a stochastic neuronal field with piecewise linear gain functions *f*(*u*) as in Eq. (3) and different slopes *γ*. Error bars, standard deviation of *T*_Down_.

Stepwise gain function *f*(*u*) = Θ(*u* − *h*) with threshold *h* ∈ (0, 1), allows to have two homogeneous fixed point of the dynamics (1) 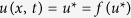 at high (Up, 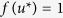) and low (Down, 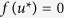) activity levels. This *f*(*u*) overlooks the possibility of having Down states with different attractive forces. Indeed, the input-output gain function measured in biological neurons^21^,^22^,^23^, and accurately characterized by the sigmoidal function *f*_*LIF*_(*u*) (Fig. 1a) of leaky integrate-and-fire (LIF) neuron models^24^,^25^, allows to have positive firing rates even for small input currents, which is not the case for the stepwise gain function when *u* < *h*. This is due to a non-vanishing steepness 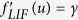 of the gain function in the Down state (*u* → 0). Such amplification of the synaptic input can be increased by strengthening the recurrent connectivity *C*_Rec_, which in turn is almost linearly related to *γ*^26^,^27^ (Fig. 1b). A steeper *f*_*LIF*_(*u*) characterizes a more excitable network, as in this case the probability to escape from the Down state is higher due to activity fluctuations (Fig. 1c). As a result, residence times in the Down state are shortened by increasing *γ*^26^. As mentioned above, this is expected to influence the propagation properties of wavefronts in neural fields endowed with such self-excitation.

**Figure 2:**
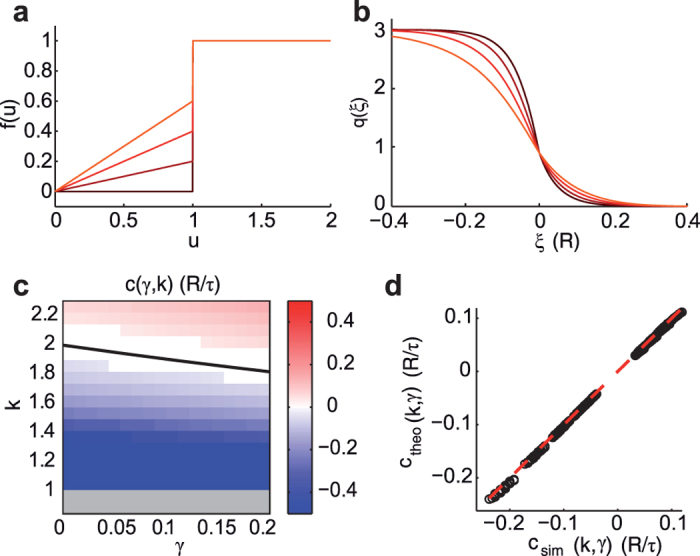
Wavefront modulation by local Down state stability (γ). (**a**) Input-output gain function *f*(*u*) for increasing *γ* (from black to red), and decreasing Down state stability. (**b**) Activation wavefronts *q*(*ξ*) corresponding to the *f*(*u*) in (**a**), numerically evaluated by solving iteratively Eq. (4). Synaptic couplings are *k* = 3 and *k*_loc_ = 0. (**c**) Wave speed *c* varying *γ* and *k* estimated from numerical simulations. White area, 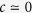 as being speed too small for numerical evaluation. Gray strip at *k* < 1, no waves occur. Black line, *k*_0_(*γ*) where *c*(*γ*, 0, *k*_0_) = 0 from Eq. (6). (**d**) Match between *c* from simulations in (**c**) (circles) and *c*(*γ*, 0, *k*) from Eq. (6) (dashed line).

In order to include this modulation of the Down state stability by changing the gain function slope *γ*, we introduce the following novel gain function


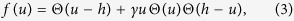


shown in Fig. 2a for three different values of *γ*. Although this piecewise linear function may appear as a rather rough approximation, it allows us to capture the dynamical features highlighted in this section. In fact, when activity fluctuations are taken into account in a neural field with such a *f*(*u*) (see below for details), Down state durations are progressively shortened by increasing *γ* (Fig. 1d), which in turn effectively modulates the stability of the low-activity state. For all these reasons in what follows we will refer to *γ* as both local excitability and Down state stability.

In this framework, activation (Down-to-Up) wavefronts can be worked out by splitting r.h.s. of Eq. (2) as


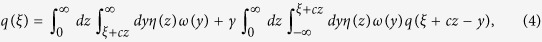


where as connectivity kernel we introduce


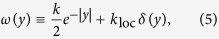


aiming at modeling the natural propensity of the cortical tissue to organize in networks of modules with synapses clustered in space^28^,^29^,^30^,^31^. Here, *δ*(*x*) is the Dirac’s delta, while *k*_*loc*_ and *k* are the strengths of the local self-coupling and the lateral nonlocal connectivity, respectively. We finally remark that, the gain function *f*(*u*) in Eq. (3) together with the local component of *ω*(*y*) describe the cortical circuitry at a spatial scale of few hundreds of microns. On the other hand, the nonlocal connectivity covers wider regions of the cortical surface which can be several millimeters long, thus making apparent the multiscale organization of the neural field under investigation here.

An approximated solution for small *γ* of Eq. (4) can be computed recursively feeding the equation r.h.s. with *q*(*ξ*) obtained in previous step. As starting wavefront we use the 0-th order approximation (*γ* = 0), which for *ξ* > 0 is *q*_0_(*ξ*) = *he*^−*ξ* ^4,5. Successive iterations provide converging higher order terms in *γ*, which can be numerically evaluated (Fig. 2b), see Supplementary Materials for additional methodological details. As a result, destabilizing Down states by increasing *γ* brings more smoothed wavefronts. A reasonable expectation is also that propagation speed changes with *γ*. To investigate this aspect, we looked for those conditions ensuring *q*(0) = *h* provided that *q*(*ξ* < 0) > *h* and *q*(*ξ* > 0) > *h*^4^,^5^, as *h* identifies the onset of an activation wavefront being *f*(*u*) = 1 if *u* > *h*. For 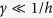, the first-order approximated expression for *q*(*ξ*) is *q*_1_(*ξ*) = *h*e^−*ξ*^ (1 + *γξ*) for *ξ* ≥ 0, as it results from Eq. (4) by setting in its r.h.s. *q*(*ξ*) = *q*_0_(*ξ*). By imposing *q*_1_(0) = *h*, two separate expressions for positive and negative wave speeds result:


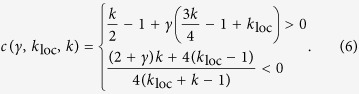


Here *h* = 1 is taken as unit measure for the synaptic current *u* and the speed solution is correct up to 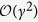. The stability of the solution has been checked in simulation.

When *c* > 0, the activation wavefront propagates towards increasing *x*, such that progressive transitions from the Down to the Up state take place, eventually bringing to the activation of the whole network. The opposite occurs for negative speeds (*c* < 0), such that Up-to-Down transitions progressively take place at increasing *x*, widening in time the inactivated region of the neural field.

To focus on the role of *γ*, we first neglect self-coupling as local feature of the field (*k*_loc_ = 0). In this case, for *k* > 1 the homogenous Up and Down attractor states are both stable. Wavefronts with stable shape can propagate towards decreasing (*c* < 0) or increasing *x (c* > 0) if *k* is smaller or larger than *k*_0_(*γ*) ≡ 4/(2 + *γ*), leading to the progressive onset of Up or Down states, respectively (Fig. 2c). Interestingly, the border *k*_0_(*γ*) where *c* = 0 decreases for increasing *γ*, highlighting the link between local stability features and the properties of nonlocal spatiotemporal patterns. Stable propagation of fronts cannot be found if *k* < 1, as the Up state is no more a fixed point. Although approximated, the speed predicted by Eq. (6) displays a remarkable agreement with simulations (Fig. 2d).

### Wave speed hysteresis due to inhomogeneous local excitability

The cortical tissue is not a homogeneous excitable medium. This diversity is reflected in the complex cytoarchitectonic organization of the brain, where size and shape of cells, together with their densities vary and fluctuate across areas and locations both in depth and along the surface of the cortex^32^. A structural variability which is reflected also in the level of heterogeneity of the nonlinear dynamics expressed by local cell assemblies both *in vivo*^33^ and *in vitro*^13^.

Inhomogeneities in neural field models have been widely investigated with different approaches^34^,^35^,^36^. Many of these studies focused on the resonant properties of the network dynamics fluctuating around a steady state of brain activity. The aim in this case was to model the power spectral densities of EEG signals recorded from the scalp of subjects at rest. In this framework, distributions in space of transmission delays^37^,^38^ and asymmetric connectivity kernels^39^,^40^ were taken into account. On the other hand, other studies investigated the impact on propagating fronts in heterogeneous media, mainly focusing on the spatial modulation of specific synaptic connectivity kernels. More specifically, only periodic^41^,^42^,^43^,^44^ and slow-varying aperiodic^45^ modulations were taken into account, aiming at modeling the columnar organization of the cortex and the slow variations in space of the neural medium features, respectively.

**Figure 3:**
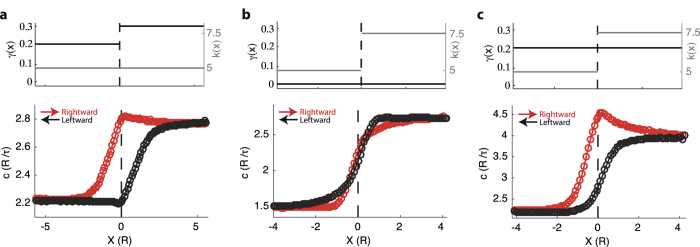
Wave speed hysteresis across the interface between neural fields. (**a**) Bottom, speed *c*(*x*) of the Up state wavefronts propagating from left to right (red) and right to left (black), respectively, in a simulated neural field with inhomogeneous slope *γ*(*x*) of the gain function in *u* = 0. Top, implemented stepwise *γ*(*x*) at the interface *x* = 0 changing from *γ*^−^ = 0.2 to *γ*^+^ = 0.3 (black), and constant strength of nonlocal connectivity strength *k*(*x*) = 5 (gray). Neural field parameters as in Fig. 1, with *k*_loc_ = 0. (**b**,**c**) Wavefront speed *c*(*x*) in opposite directions as in (**a**) for a neural field in which now the connectivity kernel *ω*(*x*) has a sudden change around *x* = 0 of the nonlocal connectivity from *k* = 5 to *k* = 7.5 (top, gray). In (**b**) the local excitability is removed – *γ*(*x*) = 0 – making inactive the Down state (*f*(*u* < *h*) = 0), while in (**c**) *γ*(*x*) = *γ*^−^ = 0.2 (top, black).

Here we contribute to this modeling enrichment by investigating how a spatial modulation of the gain function *f*(*u*), rather than the connectivity kernel, may affect the propagation of activity waves. Differently from other studies, we firstly take into account a spatial modulation with a sudden variation in space of the local excitability modulated by *γ*. More specifically, we consider two interfaced homogeneous neural fields with different excitability, such that Down state stability *γ*(*x*) is location-dependent as


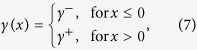


with *γ*^+^ > *γ*^−^ (Fig. 3a, top). In this case the strength *k* of lateral connectivity does not change in space. As expected, asymptotic speeds *c*(*x* → ±∞) = *c*^±^ are those predicted by Eq. (6) (Fig. 3a) such that *c*^+^ > *c*^−^. Nevertheless, around the interface *x* = 0 the wave speed measured from simulations displays an intriguing hysteresis. In fact, the speed is different depending on the direction of propagation, such that activation fronts at the same *x* move faster when waves initiate in the less excitable region, while the opposite occurs when wavefronts propagate starting from the other side. To understand how such speed hysteresis arises, we first remark that when neural field activation travels rightward an Up high-firing state is present only at *x* < 0, the speed *c*_*R*_(*x*) is expected to increase before the activity spreads through the more excitable section of the network. The opposite occurs for leftward traveling wavefronts, such that their speed *c*_*L*_(*x*) slows down well before the excitability interface is crossed. So one might argue that speeds around the interface will cross each other such that 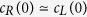, and no hysteresis would be visible. But this is not the case, indeed the wavefronts traveling in opposite directions will have different speeds in *x* = 0, as the input received is due to two different activity distribution across the network. More specifically, leftward waves at the passage through *x* = 0 see an activity *f*(*u*) = 1 from the network at *x* > 0, while on the other side they find a not quiescent Down state with firing rate *γ*^−^ *u* ≥ 0. On the other hand, rightward waves crossing *x* = 0 see the same activity *f*(*u*) = 1 now at *x* < 0, while from section *x* > 0 a more active Down state *γ*^+^ *u* ≥ 0 is found. This explains why *c*_*R*_(0) > *c*_*L*_(0), a speed difference which has to increase with the excitability jump *γ*^+^ − *γ*^−^. This kind of wave speed hysteresis is rather unexpected, as we are not considering any spatial asymmetry in the connectivity kernel *ω*(*x*), which in turn might trivially give rise to different speeds when propagation is in opposite directions.

To test the hypothesized essential role of *γ* for having the speed hysteresis, we considered an alternative situation in which by keeping *γ*(*x*) = 0 the inhomogeneity of the medium is due to a position-dependent connectivity kernel *ω*(*x, y*) obtained by extending the translation invariant *ω*(*x*) in Eq. (5):


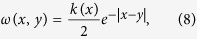


with *k*_loc_ = 0 and a connectivity strength *k*(*x*) depending on location *x*. Simulations of a neural field with a step-wise *k*(*x*) shown in Fig. 3b do not display any apparent hysteresis loop of *c*(*x*). In the same network hysteresis reappears only when a large enough *γ* is reintroduced (Fig. 3c), which further supports the hypothesis of a nontrivial interplay between local (*γ*) and nonlocal (*k*) features of neural fields: a multiscale description needed in order to capture the full dynamical richness of the biological counterpart.

**Figure 4:**
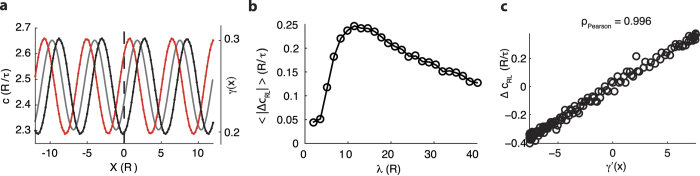
Wave speeds depend on the direction of propagation in presence of local excitability gradients. (**a**) Sinusoidal modulation of the local excitability (*γ*(*x*), gray) of the simulated neural fields superimposed to the speed *c*_*R*_(*x*) and *c*_*L*_(*x*) of the waves traveling from left to right (black) and in the opposite direction (red), respectively. (**b**) Mean of the absolute value of the speed difference Δ*c*_*RL*_ = *c*_*R*_ − *c*_*L*_ as a function of the size *R* of the connectivity kernel *ω*(*x*). (**c**) Correlation between speed difference Δ*c*_*RL*_(*x*) and local excitability gradient *γ*′(*x*).

Finally, we investigated whether speed hysteresis is a rather general feature, i.e. it does not emerge only in presence of a discontinuity of the otherwise constant excitability. To this purpose we modulated the Down state stability sinusoidally as 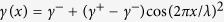, where *λ*/2 is the spatial oscillation period, while *γ*^+^ and *γ*^−^ are the same as above. As shown in Fig. 4a, hysteresis is apparent also in this case as leftward and rightward propagation speed (*c*_*L*_(*x*) and *c*_*R*_(*x*), respectively) are not in phase with *γ*(*x*). As one can expect, both this shift in space and the amplitude of speed oscillations depend on the wavelength *λ*. Indeed, computing in simulation the mean of the absolute value of the speed difference 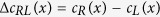, an optimal *λ* exists (Fig. 4b). This emerges from the fact that in the limit 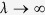 the neural field tends to have a homogenous excitability when no hysteresis occurs and 〈|Δ*c*_*RL*_|〉 → 0. The same happens if 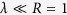 as in this case the connectivity kernel average out the excitability modulation bringing to a homogenous neural field with effective *γ* = *γ*^−^ + (*γ*^+^ − *γ*^−^)/2, i.e. the spatial average of *γ*(*x*). Both these limits are those usually investigate in the literature focusing on waves traveling in slow- and fast- varying inhomogeneous neural fields^41^,^45^, where to the best of our knowledge no speed hysteresis has been reported. Simulations on neural fields with such a sinusoidal local excitability allowed us to uncover another interesting relationship. Indeed, we found a tight linear correlation between speed difference Δ*c*_*RL*_(*x*) and the gradient *γ*′(*x*) of the Down state stability (Fig. 4c). More specifically, 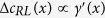 such that the larger is the variation of the local excitability, the more prominent is the difference between speed in the two propagation modes. This result is amenable to be experimentally tested and recent observations in cortical slices with slow-wave activity have provided a strong evidence of a similar linear relationship^13^.

### Diverse roles of local and lateral connectivity

**Figure 5:**
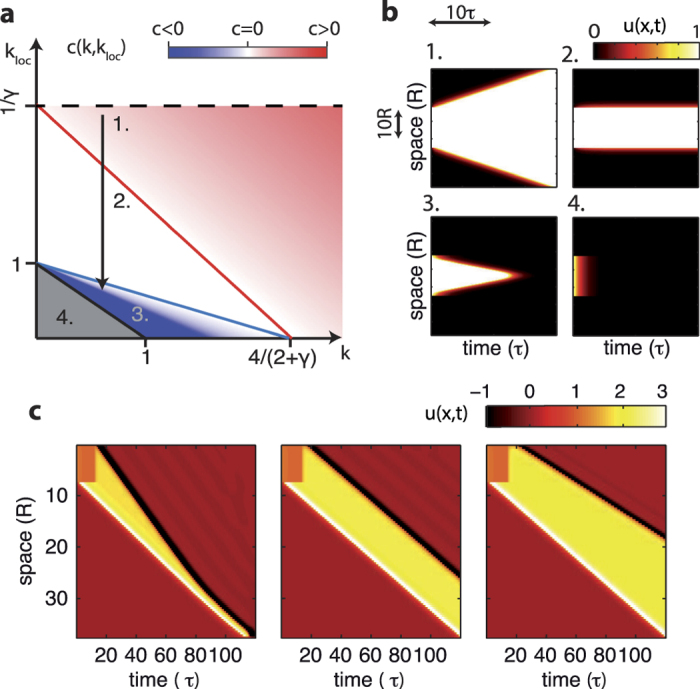
Spatiotemporal patterns of activity are governed by the interplay between local and nonlocal features. (**a**) Wave speed *c* in the diagram (*k, k*_loc_) with four phases highlighted: traveling waves with positive (phase 1) and negative (phase 3) speed; stable activity patterns occur (phase 2, *c* = 0); and no waves and spatial patterns can be self-sustained (phase 4). Above the dashed line the Down state is no more stable and stable front propagation solutions do not exist. (**b**) Examples of spatiotemporal patterns from neural field simulations for the phases in the diagram (**a**). Parameters: *γ* = 0.1, *k*_loc_ = 0.75, *k* = {0.1, 0.35, 1, 3} for phases from 4 to 1, respectively. Local activity varying in time is color coded. (**c**) Propagating Up states in simulated neural field with spike-frequency adaptation (Eq. (9)) and different local self-excitation. From left to right *k*_loc_ = {3.3, 3.4, 3.46}, while *k* = 3. Other parameters: *γ* = 0.1, *g*_a_ = 1.5 and *τ*_a_ = 2 s.

We now take into account the excitatory self-coupling *k*_loc_ > 0 in Eq. (5). This model enrichment gives rise to an additional intermediate phase where *c* = 0 and spatial Up and Down patterns can be stably self-sustained (Fig. 5b2). Such stable activity patterns occur provided that lateral connectivity *k* is not too large and *k*_loc_ is set to be in a specific range between 0 and ∞. This region is depicted by a white area in Fig. 5a, where it is also clear that the range of *k* needed to have stable Ups and Downs widens as *k* becomes smaller. More specifically, such activity regime can be expressed by the neural field provided that 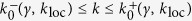 with 
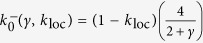
 and 
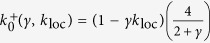
. Note that the expression for *c* = 0 from Eq. (6) only approximately recover these boundaries, as there we neglect 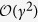 terms.

Similarly to the *k*_loc_ = 0 case, if the lateral connectivity strength *k* is not large enough, no propagating fronts exist, as the homogeneous Up state is no more a fixed point (Fig. 5b4). Having a positive local connectivity facilitates the onset of stable waves. Indeed, the lower bound for *k* depends on *k*_loc_ (*k* < 1 − *k*_loc_) and the no-waves region shrinks with increasing *k*_loc_ (Fig. 5a, gray area). On the other hand, if *k*_loc_ > 1/*γ* (Fig. 5a above the dashed line) the homogeneous Down state is still a fixed point, but it is no longer stable and thus a stable front solution does not exist. In the remaining regions, stable waves can travel. Waves with positive speed (*c* > 0, Fig. 5b1) can be found in the red shaded area of the bifurcation diagram (Fig. 5a, 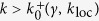). Instead, activation wavefronts traveling in the opposite direction (*c* < 0) occur in the blue shaded area of the same diagram, placed at lower values of local and nonlocal connectivity strengths 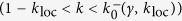. In Fig. 5a the difference of roles between *k*_loc_ and *k* is apparent. For relatively small *k* and *k*_loc_ > *k*, local activity is less prone to transition between Up and Down state, reducing the propensity to have propagating waves. The opposite occurs for strong enough lateral connectivity *k*. Phase diagram is also affected by *γ*. In fact, increasing *γ* leads to less attractive Down states, which at the same time shrinks the region with negative speeds and widens the one where *c* > 0. Besides, from Eq. (6) it turns out that *k*_*loc*_ affects only positive speed as it provides in the low activity state an excitatory feedback through *γ*. This is not the case for *c* < 0 as the wavefronts start from active states.

### Spike-frequency adaptation and wavefront synchronization

We also investigated the effect of spike frequency adaptation (SFA) on propagation. SFA is often modeled as a local activity-dependent negative feedback reducing progressively the rate of emitted spikes when firing frequency is high^46^,^47^,^48^. Its role in neural fields has been extensively investigated by including an additional inhibitory current proportional to a fatigue level *a*(*x, t*) different for each neuronal population *x* and integrating local firing rate *f*(*u*)^49^,^50^:


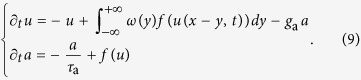


Here *g*_a_ is the strength of the negative feedback and *τ*_a_ is the relaxation time of the adaptation level *a* in the absence of spiking activity. Once a stable Up state is reached, the inhibitory feedback is −*τ*_a_ *g*_a_ *f*(*u*), which is equivalent to dampen local self-excitation as *k*_loc_ → *k*_loc_ − *τ*_a_ *g*_a_, as *f*(*u*) = 1 in this case. For this reason the characterization of the traveling wavefronts in the (*k, k*_loc_) plane may allow to grasp a qualitative picture of what is going on when SFA mechanism is taken into account. In fact, the neural field state in (*k, k*_loc_) can be roughly represented as having a downward vertical shift (arrow in Fig. 5a) leading to different possible scenarios of Up state propagation and extinction, which include traveling pulses as those investigate in refs 49,50. For large enough values of *g*_a_, Up-to-Down transitions can follow the Down-to-Up ones with a certain delay from local activation. This gives rise to a wave whose negative speed can be approximately computed by considering the above rescaling of *k*_*loc*_. Depending on the differences between the propagation speed of activation (*c*_+_ ≡ *c* > 0) and silencing (*c*_−_ ≡ *c* < 0) fronts, traveling Up states with time-varying size are thus expected. We checked this prediction by integrating numerically Eq. (9) and obtaining shrinking (Fig. 5c left), growing (Fig. 5c right) and a size-invariant Up states traveling across the neural field (Fig. 5c center). In particular, Up state shrinking can converge to a stable propagating pulse^49^,^50^, or disappear if synaptic strength is too weak (not shown).

Traveling Up states underlie slow-wave activity in the cerebral cortex of mammals during deep sleep and anesthesia^51^, and interestingly, *in vivo* Up-to-Down transitions occur more synchronously in time than Up state onset when probed simultaneously across the cortical surface^52^,^53^. The above interplay between local and nonlocal connectivity features can explain such difference provided that 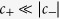. Indeed, having a fully restored neural field following a long enough Down period would imply a negligible adaptation level. Under this condition the network can have an effective *k*_loc_ so large to place the system just above the red boundary in Fig. 5a. As a result, Up state onsets would be elicited by a moderately high *γk*_loc_. In other words, *c*_+_ might be relatively small and the weakly stable Down state would be highly sensitive to endogenous noise, giving rise to a widely fluctuating advancement of the wavefront (see next Section). This could explain the asynchronous nature of the *in vivo* Up state onset. On the other hand, during the Up state, the adaptation level can be set to grow highly enough to produce a self-inhibition capable to destabilize the high-firing state, such that the effective *k*_loc_ decreases as shown by the vertical arrow in Fig. 5a. If such inhibition would be strong enough, the advancement speed *c*_−_ of the inactive region of the neural field could be so fast that the network inactivation would appear as almost simultaneous. In this way, the synchronous silencing of the cortex would result from strongly adapted neuronal assemblies which persist in their Up states only thanks to the input provided by the active nearby neurons.

### Optimal connectivity balance minimizes wavefront fluctuations

Activation wavefronts measured *in vivo* during slow-wave activity are irregular in space^54^,^55^. Motivated by this, we investigated if and how multiscale connectivity can contribute to such irregularity when activity fluctuations are taken into account. Following^56^,^57^,^58^ we extended the dynamics (1) as


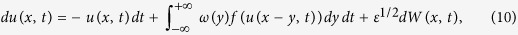


where input current *u* incorporates an additional stochastic force modeling finite-size fluctuations of activity^59^,^60^,^61^ as a Wiener process *dW*(*x, t*) uncorrelated both in space and in time: 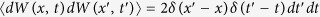. Although finite-size fluctuations are known to be activity dependent^60^,^61^, we explicitly introduce an additive noise rather than a multiplicative one to compensate the fact that the Down state of the neural field is associated to the *u* = 0 fixed point. Indeed, under this condition a noise *dW* (*x, t*) proportional to *u* would not induce any fluctuation of the input during the Down state, thus failing to effectively model biological neuronal networks. In our approach *dW* (*x, t*) aims to represent the noisy component of the local input current due to the endogenous activity fluctuations of the pre-synaptic cell assemblies.

As *γ* affects the stability of Down state through the activity amplification due to *k*_loc_, an increase of *γk*_loc_ will make input current fluctuations growing before Down-to-Up transition^26^. Here, we quantify the impact of this activity modulation on the fluctuations in time of the traveling wavefronts. Such fluctuations can be quantified exploiting the perturbative approach introduced in ref. 57 where the wave profile *u*(*x, t*) solution of Eq. (10) is decomposed in two terms:


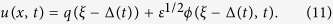


The first is the wave profile *q* obtained in absence of endogenous fluctuations (*ε* = 0), and hence solution of Eq. (4). It is displaced in time by Δ(*t*) from its uniformly translating position *ξ*. The other time-dependent term *ϕ* further shapes in time *q* in order to take into account the perturbations induced by *ε*^1/2^ *dW*. Substituting Eq. (11) into (10), the dynamics of *ϕ* results to be


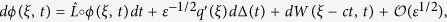


where 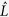 is the same linear operator as in ref. 57 (see Supplementary Material), from which the null space of the adjoint operator 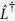 can be worked out allowing to find the solvability conditions of this equation. These can be obtained by computing the function 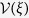 spanning such null space: 
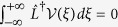
. For *k*_loc_ = 0 and 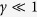 such that 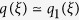, and considering only Up state onset propagation (*c* > 0), we found that 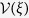 has the form:


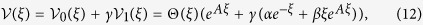


where *A* = −1/*c*_0_ with speed *c*_0_ = *k*/2 − 1 from Eq. (6) and *γ* = 0, while *α* = *k*(*k* − 2)/(*k* − 4)^2^ and *β* = *α*(*k* − 4) (2*k*^2^ − 9*k* + 8)/(*k* − 2)^3^. As in ref. 57, the solvability condition leads to the following stochastic integral equation:


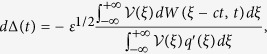


from which Δ(*t*) results to be an uncorrelated Gaussian noise with zero mean and variance 〈Δ(*t*)^2^〉 = 2*D*(*k, γ*)*t*, with diffusion coefficient


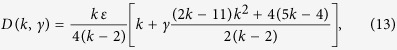


where we neglect 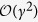 terms.

**Figure 6:**
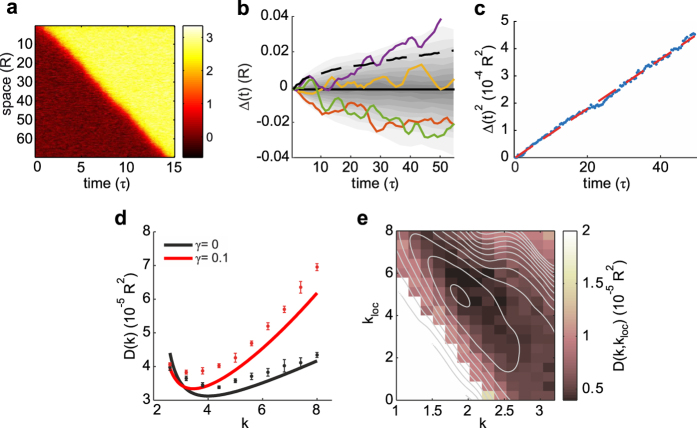
Fluctuations of activation front due to endogenous local noise. (**a**) Example simulation of stochastic front propagation, *ε*^1/2^ = 0.018, *k* = 7, *γ* = 0 and *k*_loc_ = 0. (**b**) Time course of wavefront displacement Δ(*t*) compared to the propagation expected in absence of noise. Gray shading, density of the 1000 simulated replicas. Colored curves, 4 example replicas. Dashed curve, time course of the standard deviation of the wavefront displacement 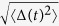. (**c**) Time course of the variance of wavefront displacement 〈Δ(*t*)^2^〉 (blue) and its linear fit (red dashed line). (**d**) Diffusion coefficient *D*(*k, γ*) for *γ* = 0 (black) and *γ* = 0.1 (red). Solid curves, theoretical value from Eq. (13). Circles and error bars, average and standard error mean from simulation (obtained over 5 measures of *D*(*k*) for each *k* value, *n* = 10000 replicas for each measure). (**e**) Estimated diffusion coefficient *D*(*k, k*_*loc*_, *γ*) in the (*k, k*_loc_) plane for *γ* = 0.1, *ε*^1/2^ = 0.035. Mean values from *n* = 2000 replicas for each (*k, k*_loc_). White region, no wave occur.

We numerically integrated neural field dynamics (10) to test this expression (Fig. 6a). Singling out the displacement Δ(*t*) of the wavefronts (Fig. 6b), we first confirmed the proportionality of its variance to *t* (Fig. 6c). The good agreement between *D*(*k, γ*) and its measured counterpart is finally shown in Fig. 6d for two example values of *γ*. As expected, increasing *γ* (from black to red), fluctuations of wavefront displacements grow. This is particularly apparent for large lateral connectivity *k*. A minimum of such variability is apparent at *k* = 4, and it shifts to lower values when *γ* increases. It is interesting to remark that, if endogenous fluctuations would be modeled by a multiplicative noise, the minimum of the wavefront variability would not exist, as in this case *D*(*k*) would decrease monotonically with *k*^57^.

To investigate whether this particular noise shaping is displayed also when local self-excitation *k*_loc_ is taken into account, we estimated *D*(*k, k*_*loc*_, *γ*) directly from simulations (Fig. 6e). As a result, a minimum *D* not only exists for *k*_loc_ > 0 but it is shifted at lower *k* when *k*_loc_ increases. More intriguingly, a global minimum emerges at 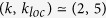. This suggests the existence of an optimal combination of local and nonlocal connectivity leading to minimally irregular waves in neural fields with local additive noise.

Here it is interesting to remark that in Eq. (13), the diffusion coefficient *D*(*k, γ*) with *k*_loc_ = 0 depends linearly on *ε*, i.e. the shape of *D* does not depend on the noise size 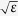, and only the structural parameters *γ* and *k* matter. For this, reason the minimum of *D*(*k, γ*) is found to be always at the same lateral connectivity strength *k* for any *ε*. We then tested in simulation whether the same occurs also for *k*_loc_ > 0 (see Supplementary Materials), finding under this condition once again no changes in the position of the *D*(*k, k*_loc_) minimum. This numerical result further corroborates the hypothesis that the optimal balance between local and lateral connectivity mentioned above is an intrinsic structural property of the network and does not depend neither on the network activity nor on the intensity 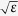 of the endogenous fluctuations.

## Discussion

Traveling waves are a nonlinear phenomenon pervasively expressed by excitable media ranging from physical to biological systems^1^,^2^,^3^,^4^,^5^. In the latter, such spatiotemporal patterns often emerge from the interactions between components differently expressed across several spatial and temporal scales^9^,^10^,^11^,^12^,^13^. Focusing on the specific example of front propagation across the cortical tissue, here we have shown the crucial role of modeling the local nonlinear dynamics of cell assemblies. The introduction of the slope *γ* of *f*(*u*) in *u* = 0 has aimed at parametrically modulate the Down state stability and has allowed to analytically derive the related wavefront properties. Once the excitability *γ* is inhomogeneous in space, a novel hysteresis phenomenon for the wave speed emerges. More specifically, at the interface between two neural fields with different Down state stability, the propagation speed is expected to be different if waves travel in opposite directions. This prediction is in agreement with recent findings in experiments with cortical slice^13^. Besides, we expect this result could provide additional insights to understand the compression phenomenon of traveling Up states crossing the border between primary (V1) and secondary (V2) visual cortex observed in anesthetized rodents^6^.

Synaptic connectivity distributed at different spatial scales is another hallmark of biological networks of neurons. We have enriched neural field models taking this into account, eventually showing that traveling waves can display qualitatively different properties. In this framework, speed and stochastic displacement of propagating wavefronts are still amenable to analytical treatment, and the theoretical results we have derived, can be of general applicability to propagation problems like spreading of epidemics or information across multiscale mobility and communication networks^62^,^63^.

Our theoretical findings provide insights into the mechanistic substrate of slow-wave activity occurring across the cerebral cortex of mammalian brains^51^. Local and nonlocal connectivity play different roles in Up state propagation which is critically governed by the subthreshold feedback of local activity proportional to *γk*_loc_. This contributes to modulate Down state stability favoring the occurrence of Down-to-Up transitions and to increase propagation speed. As in brain slices Up state onset preferentially occurs and starts propagating just below the granular layer, this might highlight a different local excitability across layers^9^,^12^,^13^. Multiscale connectivity affects also the Up state offset. Indeed, if activation is primed by synaptic self-coupling, late sustained firing in the Up state has been hypothesized to be exogenous-driven^64^, due to SFA. Hence, lateral connectivity can play a key role in determining the speed of the Up-to-Down wavefronts, which in turn can be more synchronous in time than activation fronts^52^,^53^ as we have shown here providing a mechanistic explanation of related *in vivo* experimental evidence.

Noise in the synaptic input due to the finite-size fluctuations of local cell assemblies, further emphasize the interplay between spatial scales. Up wavefronts show random displacements whose size is minimized by an optimal choice of (*k, k*_loc_). This new form of noise shaping disappears if local fluctuations are absent as in a fully quiescent Down state (see ref. 57). Intriguingly, such dampening of noise could explain the evidence found in cortical slices that activation wavefronts are maximally sensitive to the layered structure of the cortex only for a specific balance between intra- and intermodular connectivity^13^.

## Additional Information

**How to cite this article:** Capone, C. and Mattia, M. Speed hysteresis and noise shaping of traveling fronts in neural fields: role of local circuitry and nonlocal connectivity. *Sci. Rep.* **7**, 39611; doi: 10.1038/srep39611 (2017).

**Publisher's note:** Springer Nature remains neutral with regard to jurisdictional claims in published maps and institutional affiliations.

## Supplementary Material

Supplementary Information
