# Supplementary material for: Speed hysteresis and noise shaping of traveling fronts in neural fields: role of local circuitry and nonlocal connectivity
Source: Sci Rep. 2017 Jan 3;7:39611. doi: 10.1038/srep39611 (PMC5206719; doi:10.1038/srep39611)
Supplement: Supplementary Information [file srep39611-s1.pdf]

Speed hysteresis and noise shaping of traveling fronts in neural fields: role of local circuitry and nonlocal connectivity.

Cristiano Capone[1,2*], **Maurizio Mattia**[1]

1 Istituto Superiore di Sanità, Rome, Italy
2 Physics Department, Sapienza University, Rome, Italy

*cristiano0capone@gmail.com

1 Supplementary Information

1.1 Iterative evaluation for propagating front

To numerically solve self-consistent Eq. (4) of the main text to work out the stable wavefronts $q(\xi)$ when Down state stability of the network changes with $\gamma > 0$, we developed an iterative procedure. We first compute an initial 0-th order ($\gamma = 0$) guess

$$q_0(\xi, c_0) = \int_0^\infty dz \int_{\xi+cz}^\infty dy\, \eta(z)\, \omega(y)\,, \tag{1}$$

which is numerically integrated together with the condition which allows to determine the speed c_0:

$$q_0(0, c_0) = 1\,, \tag{2}$$

as described in the main text. Given this initial guess for $q(\xi, c)$ and the speed c, we recursively evaluate high-order approximations as follows

$$q_{n+1}(\xi, c_{n+1}) = \int_0^\infty dz \int_{\xi+cz}^\infty dy\, \eta(z)\, \omega(y) + \gamma \int_0^\infty dz \int_{-\infty}^{\xi+cz} dy\, \eta(z)\, \omega(y)\, q_n(\xi + cz - y, c_n)\,, \tag{3}$$

where at each step both q_{n+1} and c_{n+1} numerically evaluated to satisfy also the expression

$$q_{n+1}(0, c_{n+1}) = 1\,. \tag{4}$$

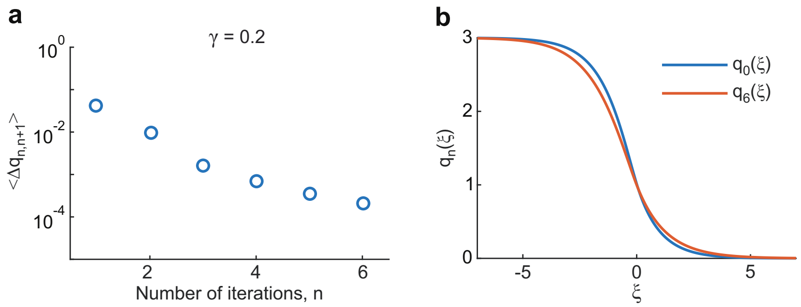

Figure 1. Iterative procedure to obtain propagating fronts for $\gamma > 0$. (**a**) Average difference between fronts of two consecutive iterations q_n and q_{n+1} with $\gamma = 0.2$. (**b**) Front at step $n = 0$ (blue) and $n = 6$ (red) for $\gamma = 0.2$.

We tested the convergence of the method by evaluating the average difference between front obtained at consecutive steps $\Delta q_{n,n+1} = \int |q_{n+1}(\xi) - q_n(\xi)| d\xi$. As shown in Fig. 1a for an example set of parameters, we found a fast reduction of the difference between consecutive q_n bringing rapidly to an accurate estimate of the asymptotic $q(\xi, c)$. For illustrative purposes, in Fig. 2b of the main text we displayed $q_6(\xi, c_6)$, as it is rather apparent from Fig. 1b that such expression closely reproduces the asymptotic $q(\xi, c)$.

1.2 Neural field simulations

Numerical simulations of neural field are performed by discretizing space and time at steps $dx = 0.1R$ and $dt = 0.3\tau$, respectively. Differential equations are numerically integrated by resorting to the Euler's method:

$$u(x_i, t+dt) = u(x_i, t) + [-u(x_i, t) + \sum_k \omega(y_k) f(u(x_i - y_k))dx]dt\,. \tag{5}$$

Integration of stochastic neural field dynamics is performed by adding white noise to the above equation:

$$u(x_i, t+dt) = u(x_i, t) + [-u(x_i, t) + \sum_k \omega(y_k) f(u(x_i - y_k))dx]dt + \varepsilon^{-\frac{1}{2}} dW(x,t)\,, \tag{6}$$

where $W(x,t)$ are different Wiener processes depending on the position x with zero mean and correlation structure

$$\langle dW(x,t)\rangle = 0 \qquad \langle dW(x,t)dW(x',t')\rangle = 2C(x-x',\lambda)\delta(t-t')dtdt'\,.$$

Cross-correlation $C(x-x',\lambda)$ decays on a spatial scale $\lambda \ll dx$, such that we can safely assume $C(x-x',\lambda) = \frac{1}{dx}$. Thus stochastic source of the input current u is

$$dW(x,t) = \sqrt{\frac{2}{dx}}\sqrt{dt}\mathscr{N}(t) \tag{7}$$

where $\mathscr{N}(t)$ is a Gaussian random variable with 0 mean and unitary variance 1.

The diffusion coefficient D has been computed as the infinitesimal variance of $\Delta(t)$:

$$D = \lim_{t\to\infty}\left\langle \frac{\Delta(t)^2}{2t} \right\rangle\,. \tag{8}$$

Average $\langle . \rangle$ was computed from N_{rep} statistical independent replicas of the same stochastic neural field (the number is specified in the main text). Wavefront position displacement $\Delta(t)$ was computed as the difference between the single replica position $x(t)$ of the front and its average $\langle x(t)\rangle$ across replicas. The front position is defined as $u(x(t),t) = h = 1$, namely the position where the current u crossed for the first time the value $h = 1$.

1.3 Null space and effective diffusivity

As pointed out in the main text the displacement $\Delta(t)$ between stochastic and deterministic front at time t can be worked out from the following stochastic integral equation:

$$d\Delta(t) = -\varepsilon^{1/2}\frac{\int_{-\infty}^{+\infty}\mathscr{V}(\xi)\,dW(\xi - ct, t)\,d\xi}{\int_{-\infty}^{+\infty}\mathscr{V}(\xi)\,q_1'(\xi)\,d\xi}\,,$$

where the approximated expression $q_1(\xi)$ of the activation wavefront $q(\xi)$ in which $\mathscr{O}(\gamma^2)$ terms are neglected. From this, $\Delta(t)$ results to be an uncorrelated Gaussian noise with zero mean and variance $\langle\Delta(t)^2\rangle = 2D(k,\gamma)t$, with coefficient of diffusion

$$D(k,\gamma) = \varepsilon\frac{\int_{-\infty}^{\infty}\mathscr{V}(\xi)^2 d\xi}{[\int_{-\infty}^{\infty}\mathscr{V}(\xi)q_1'(\xi)d\xi]^2} \tag{9}$$

The function $\mathscr{V}(\xi)$ spanning the null space of the adjoint operator $\hat{L}^\dagger$, can be found by solving the following integro-differential equation

$$\hat{L}^\dagger \circ \mathscr{V}(\xi) = 0$$

which for the specific case treated here, leads to the equation

$$c\frac{d\mathscr{V}(\xi)}{d\xi} + \mathscr{V}(\xi) = f'(q_1(\xi))\int_{-\infty}^{\infty}\omega(\xi - \xi')\mathscr{V}(\xi')d\xi'\,,$$

where we took into account that the linear operator $\hat{L}$ is

$$\hat{L}\circ A(\xi) = c\frac{dA(\xi)}{d\xi} - A(\xi) + \int_{-\infty}^{\infty}\omega(\xi-\xi')f'(q_1(\xi'))A(\xi')d\xi'\,.$$

Here $A(\xi)$ is a generic function belonging to $L_2(R)$ (see Ref.[1]). The adjoint operator $\hat{L}^\dagger$ is on the other hand defined as

$$\int_{-\infty}^{\infty} B(\xi)\hat{L}\circ A(\xi)d\xi \equiv \int_{-\infty}^{\infty}\left[\hat{L}^\dagger\circ B(\xi)\right]A(\xi)d\xi\,,$$

where $B(\xi)$ is another arbitrary integrable function.

Being in our case $f(q) = \Theta(q-h)+\gamma q\Theta(h-q)\Theta(q)$, its first derivative is

$$f'(q) = (1-\gamma)\frac{\delta(\xi)}{|q'(0)|}+\gamma\Theta(h-q)\,,$$

where we used $\Theta(q) = 1$ because $q(\xi) > 0$ for any ξ value, if $q(\xi)$ is the deterministic solution. Finally, we obtain

$$c\frac{d\mathscr{V}(\xi)}{d\xi}+\mathscr{V}(\xi) = \delta(\xi)\frac{(1-\gamma)}{|q_1'(0)|}\int_{-\infty}^{\infty}\omega(\xi')\mathscr{V}(\xi')d\xi'+\gamma\Theta(h-q1)\int_{-\infty}^{\infty}\omega(\xi-\xi')\mathscr{V}(\xi')d\xi'\,,$$

As pointed out in the main text the approximated activation wavefront $q_1(\xi)$ result to be

$$q_1(\xi) = e^{-\xi}(1+\gamma\xi) \qquad \text{for } \xi > 0\,, \tag{10}$$

while the expression for $\xi < 0$ is not reported here being unnecessarily complicated. It is anyway important to remark that its derivative is continuous around $\xi = 0$:

$$\lim_{\xi\to 0^+} q_1(\xi) = \lim_{\xi\to 0^-} q_1(\xi) = \gamma-1\,,$$

such that $|q_1'(0)| = 1-\gamma$. Recalling that the connectivity kernel $\omega(y) = \frac{k}{2}e^{-|y|}$ for $k_{\text{loc}} = 0$, we finally obtain

$$c\frac{d\mathscr{V}(\xi)}{d\xi}+\mathscr{V}(\xi) = \delta(\xi)\int_{-\infty}^{\infty}\omega(\xi')\mathscr{V}(\xi')d\xi'+\gamma\Theta(h-q1)\int_{-\infty}^{\infty}\omega(\xi-\xi')\mathscr{V}(\xi')d\xi'\,.$$

This equation can be perturbatively solved once again neglecting the $\mathscr{O}(\gamma^2)$ terms, and assuming $\mathscr{V}(\xi)$ to have the following form

$$\mathscr{V}(\xi) = \mathscr{V}_0(\xi)+\gamma\mathscr{V}_1(\xi) = \Theta(\xi)\left(e^{A\xi}+\gamma(\alpha e^{-\xi}+\beta\xi e^{A\xi})\right)\,. \tag{11}$$

Also the speed has to be developed in Taylor's series and approximated as

$$c = c_0+\gamma c_1\,. \tag{12}$$

The zero-th order in γ of this equation is

$$c_0\frac{d\mathscr{V}_0(\xi)}{d\xi}+\mathscr{V}_0(\xi) = \delta(\xi)\int_{-\infty}^{\infty}\omega(\xi')\mathscr{V}_0(\xi')d\xi'\,,$$

and by replacing $\mathscr{V}_0(\xi) = \Theta(\xi)e^{A\xi}$ we have

$$c_0(\delta(\xi)e^{A\xi}+\Theta(\xi)Ae^{A\xi})+\Theta(\xi)e^{A\xi} = \delta(\xi)\int_{-\infty}^{\infty}\omega(\xi')\Theta(\xi)e^{A\xi'}d\xi'\,,$$

which simplifies into

$$\delta(\xi)c_0+\Theta(\xi)c_0Ae^{A\xi}+\Theta(\xi)e^{A\xi} = \delta(\xi)\int_0^{\infty}\omega(\xi')e^{A\xi'}d\xi'\,.$$

Computing the integral the resulting expression

$$\delta(\xi)c_0+\Theta(\xi)c_0Ae^{A\xi}+\Theta(\xi)e^{A\xi} = \delta(\xi)\frac{k}{2(1-A)}\,,$$

allows to work out the coefficients c_0 and A by imposing the polynomial identity:

$$\begin{cases} c_0 &= \dfrac{k}{2}-1 \\ A &= \dfrac{2}{2-k} \end{cases} . \tag{13}$$

On the other hand, the first-order part of the equation for $\mathscr{V}$ is

$$c_0\frac{d\mathscr{V}_1(\xi)}{d\xi}+c_1\frac{d\mathscr{V}_0(\xi)}{d\xi}+\mathscr{V}_1(\xi)=\delta(\xi)\int_{-\infty}^{\infty}\omega(\xi')\mathscr{V}_1(\xi')d\xi'+\Theta(h-q1)\int_{-\infty}^{\infty}\omega(\xi-\xi')\mathscr{V}_0(\xi')d\xi',$$

which by replacing $\mathscr{V}_0(\xi)=\Theta(\xi)e^{A\xi}$ and $\mathscr{V}_1(\xi)=\Theta(\xi)(\alpha e^{-\xi}+\beta e^{A\xi})$ expands as

$$c_0\left[\delta(\xi)(\alpha e^{-\xi}+\beta\xi e^{A\xi})+\Theta(\xi)(-\alpha e^{-\xi}+\beta A\xi e^{A\xi}+\beta e^{A\xi})\right]+c_1\left[\delta(\xi)e^{A\xi}+\Theta(\xi)Ae^{A\xi}\right]+\Theta(\xi)(\alpha e^{-\xi}+\beta\xi e^{A\xi})$$
$$=\delta(\xi)\int_{-\infty}^{\infty}\omega(\xi')\Theta(\xi)(\alpha e^{-\xi'}+\beta\xi e^{A\xi'})d\xi'+\Theta(h-u)\int_{-\infty}^{\infty}\omega(\xi-\xi')\Theta(\xi)e^{A\xi'}d\xi'$$

Now taking into account the properties of the function $\delta(\xi)$ and considering $A\,c_0=-1$, this expression can be rewritten as

$$c_0\left[\delta(\xi)\alpha+\Theta(\xi)(-\alpha e^{-\xi}+\beta e^{A\xi})\right]+c_1\left[\delta(\xi)+\Theta(\xi)Ae^{A\xi}\right]+\Theta(\xi)\alpha e^{-\xi}=$$
$$\delta(\xi)\alpha\int_0^{\infty}\omega(\xi')e^{-\xi'}d\xi'+\delta(\xi)\beta\int_0^{\infty}\omega(\xi')\xi e^{A\xi'}d\xi'+\Theta(h-q1)\int_0^{\infty}\omega(\xi-\xi')e^{A\xi'}d\xi',$$

which by solving the integrals on the right hand side it reduces to

$$\delta(\xi)c_0\alpha+\Theta(\xi)(-\alpha c_0e^{-\xi}+\beta c_0e^{A\xi})+\delta(\xi)c_1+\Theta(\xi)c_1Ae^{A\xi}+\Theta(\xi)\alpha e^{-\xi}=$$
$$\delta(\xi)\alpha\tfrac{k}{4}+\delta(\xi)\beta\tfrac{k}{2(1-A)^2}+\Theta(h-q1)(-\tfrac{k}{2(A+1)}e^{-\xi}+\tfrac{k}{(1-A^2)}e^{A\xi}).$$

Once again, by imposing the polynomial identity, the following system of equations results

$$\begin{cases} -\alpha c_0+\alpha &= -\dfrac{k}{2(A+1)} \\ \beta c_0+c_1A &= \dfrac{k}{1-A^2} \\ c_0\alpha+c_1 &= \alpha\dfrac{k}{4}+\beta\dfrac{k}{2(1-A)^2} \end{cases} \tag{14}$$

from which the unknown coefficients α, β and c_1 can be worked out

$$\begin{cases} \alpha &= \dfrac{(k-2)k}{(k-4)^2} \\ \beta &= \dfrac{k\left(2k^2-9k+8\right)}{(k-4)(k-2)^2} \\ c_1 &= \dfrac{3}{4}k-1 \end{cases} . \tag{15}$$

This completely defines $\mathscr{V}(\xi)$ approximated at the first-order in γ in the Eq. (11). We remark that c_0 and c_1 are both in agreement with the expression for the positive speed found in the main text.

Given $\mathscr{V}(\xi)$ the coefficient of diffusion $D(k,\gamma)$ can be finally computed from Eq. (9), which neglecting $\mathscr{O}(\gamma^2)$ finally recover

$$D(k,\gamma)=\frac{k\varepsilon}{4(k-2)}\left[k+\gamma\frac{(2k-11)k^2+4(5k-4)}{2(k-2)}\right], \tag{16}$$

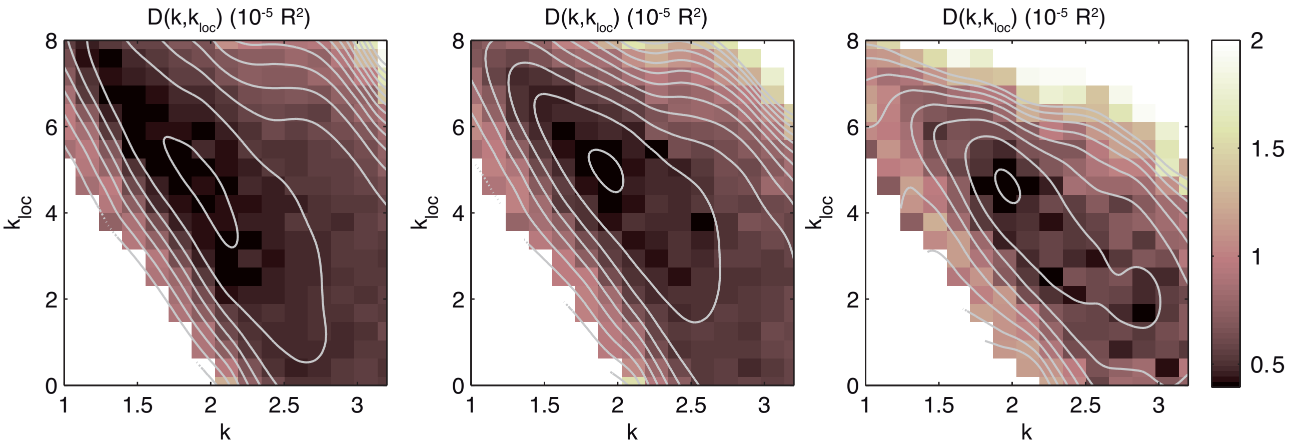

Figure 2. Invariance of the minimum of the wavefront diffusivity $D(k, k_{\text{loc}})$ with respect to the modulation of the endogenous noise intensity ε. Numerical estimations are in the plane (k, k_{loc}) for different ε values: $\varepsilon_1 = 0.7 \times 10^{-3}$, $\varepsilon_2 = 1.2 \times 10^{-3}$ and $\varepsilon_3 = 2.8 \times 10^{-3}$ displayed from the left to the right panel, respectively. The intermediate value ε_2 is the same as the one shown in Fig. 5d of the main text.

1.4 Numerical Diffusivity estimation.

As shown in Eq. (16), the analytical results suggest that the wavefront diffusivity $D(k, k_{\text{loc}})$ depends linearly on ε when $k_{\text{loc}} = 0$. This means that the shape of $D(k) = D(k, 0)$ is independent from ε and only the structural parameters γ and k matter. As a result, minimum of $D(k)$ is expected to be found always at the same lateral connectivity strength k. The same is not guaranteed if $k_{\text{loc}} > 0$, but we conjecture that the independence from ε holds also when local connectivity is incorporated. We tested such hypothesis in simulation by checking such invariance for different values of ε (Fig. 2). Although the magnitude of $D(k, k_{\text{loc}})$ changes with ε as expected, the position of the minimum in the plane (k, k_{loc}) does not change, confirming that what found for $k_{\text{loc}} = 0$ holds also for the more general case with $k_{\text{loc}} > 0$. Hence, the optimal balance between local and lateral connectivity minimizing the fluctuations of the propagating wavefronts appears to be an intrinsic structural property, i.e. it does not depend neither on the network activity nor on the intensity ε of the endogenous fluctuations.

References

1. P. C. Bressloff and M. A. Webber, SIAM J. Appl. Dyn. Syst. **11**, 708 (2012).
